# The human tickle response and mechanisms of self-tickle suppression

**DOI:** 10.1098/rstb.2021.0185

**Published:** 2022-11-07

**Authors:** Sandra Proelss, Shimpei Ishiyama, Eduard Maier, Matthias Schultze-Kraft, Michael Brecht

**Affiliations:** ^1^ Humboldt-Universität zu Berlin, 10117 Berlin, Germany; ^2^ Bernstein Center for Computational Neuroscience Berlin, 10115 Berlin, Germany; ^3^ NeuroCure Cluster of Excellence, Charité–Universitätsmedizin Berlin, 10117 Berlin, Germany; ^4^ Institut für Pathophysiologie, Universitätsmedizin der Johannes-Gutenberg-Universität Mainz, 55128 Mainz, Germany; ^5^ Charité–Universitätsmedizin Berlin, corporate member of Freie Universität Berlin, Humboldt-Universität zu Berlin, and Berlin Institute of Health, Berlin, Germany

**Keywords:** ticklishness, gargalesis, self-tickle suppression, sensory attenuation, self-touch

## Abstract

A tickle is a complex sensation: it occurs in response to touch but not unequivocally so, and makes us laugh albeit not when we self-tickle. We quantified human ticklishness by means of physiological, visual and acoustic measures alongside subjective reports, and assessed mechanisms of self-tickle suppression. Tickle responses arose faster than previously reported as changes in thoracic circumference and joyous facial expressions co-emerge approximately 300 ms after tickle onset and are followed by vocalizations starting after an additional 200 ms. The timing and acoustic properties of vocalizations tightly correlated with subjective reports: the faster, louder and higher-pitched participants laughed, the stronger they rated the experienced ticklishness. Externally evoked ticklishness is reduced by simultaneous self-tickling, whereby self-touch evokes stronger suppression than sole self-tickle movement without touch. We suggest that self-tickle suppression can be understood as broad attenuation of sensory temporally coincident inputs. Our study provides new insight on the nature of human ticklishness and the attenuating effects of self-tickling.

This article is part of the theme issue ‘Cracking the laugh code: laughter through the lens of biology, psychology and neuroscience’.

## 1. Introduction

We might rub and scratch or laugh and wriggle in response to ticklish sensations. In fact, ticklishness describes two very different percepts: one follows touch that is light and feathery, like a spider crawling upon one's skin (termed knismesis), the other touch that is heavy and rhythmic, and commonly associated with playful and interactive behaviour (termed gargalesis; [1]). While knismesis is readily elicited on any part of the body, and may be self-evoked, gargalesis is invariably more intricate. It occurs only in response to touch at certain parts of the body, is dependent on mood and context and generally not evocable through self-touch [2]. It is the only form of touch that provokes laughter, placing it in a unique position of the behavioural repertoire.

Efforts to categorize ticklish responses were undertaken by Hall and Alliń, who conducted a vast survey on ticklishness in children, albeit without considering the distinction of gargalesis and knismesis they went on to coin [1]. They noted that ticklishness varies as a function of body area with the sole of the feet ranking the highest, followed by armpit, neck and chin. Since then, others have identified similar ticklish body sites in adults, if not in the same order and reported without clear methodological description [3,4]. Alas, the nomenclature of ticklishness in human research is frequently used indiscriminately, to describe either knismesis or gargalesis [3]. Arguably, investigations into human tickling often evoke knismesis responses by utilization of equipment such as soft foam or brush to elicit light stimulation (see [5–7]).

Gargalesis is not unique to humans but also occurs in other animals including primates [8,9] and rats [10,11]. Rats emit vocalizations associated with positive affect and reward valence during tickling by humans [10,12]. Similar to humans, response to tickling in rats is dependent on body parts and mood [10–13]. Yet, while physiological parameters during tickling have been studied extensively in rats [14], comparative studies in humans are missing.

A remarkable feature of tickle perception is the so-called ‘tickle effect’, referring to the well-known observation that we cannot tickle ourselves [15]. Within somatosensory systems, self-elicited tactile stimulation is perceived with less intensity than the same stimulation applied externally [16]. In-line, self-touch (presumably knismesis) is reliably rated as less ‘tickly’, ‘intense’ or ‘pleasant’ than externally evoked touch sensation [5,6,17]. The prevailing assumption is that lessened perception of a self-tickle is based on the precise predictability of the sensory consequences of self-generated actions [18–22].

In order to derive a framework of gargalesis and self-touch mediated sensory suppression in humans a physiological characterization is needed. Here, we (i) conducted a time-series analysis of psychophysiological mechanisms (i.e. thoracic circumference, facial expression, vocalizations) during a tickle response and (ii) related these measures to the subjective experience of ticklishness. (iii) Next, we investigated self-touch mediated sensory suppression during coinciding allo- and self-tickling and explored the respective contributions of self-generated motion versus touch in the suppression of ticklishn­ess.

## 2. Methods

### (a) Participants

Twelve participants (eight female; mean age 29.7 years, s.d. 3.65 years) joined the experiment in pairs to ensure familiarity, each taking on the role of ticklee and tickler in order of their choosing. Data from one participant had to be excluded due to technical issues during the recording. Participants were recruited from within the research group and the social circle of members of the group. This approach was chosen based on observations in rats showing high mood-dependence of ticklishness and ticklishness suppression during anxiogenic scenarios [11] as well as recommendation of past research to use an approach that enhances familiarity during tickling for further studies [23].

### (b) Experimental set-up

Participants were seated comfortably on a chair and fixated on a fixation cross in proximity to the recording set-up. Two cameras (GoPro Hero Black, full HD, 240 fps; GoPro, Inc, San Mateo, CA, USA) recorded tickle events and responses of the participants, with one directed at the participants' frontal view and one at the participants' right foot. The latter was required to film touch onsets on the sole of the foot that was not visible on the frontal-facing camera. Vocalizations were recorded via a microphone (Rode VideoMicro) placed adjacent to the front camera at a distance of 65 cm from the participant. A respiratory belt transducer (AD Instruments, TN1132/ST) was used to measure changes in chest diameter in response to tickling.

### (c) Experimental design

Ticklers were instructed to keep the tickling short, using their thumb, index and middle finger, and to limit tickling to one tickle event per trial. Ticklees were told to act as naturally as possible, that is not to force but also not to withhold laughter. The ticklee was always approached from behind as to avoid knowledge of the exact time point of the tickle, and tickled exclusively on the right side of the body. After completion of each tickling event participants rated the experienced ticklishness on a Likert-type scale from 0 to 10, with 0 equating to ‘not ticklish at all’ and 10 equating to ‘highly ticklish’. Participants were advised that absolute values were not of importance but rather to keep the scaling of scores consistent across events and trials. The experiment comprised two main parts, with addition of an intermediate supplementary part. The three parts (detailed below) were always conducted in the same order for all participants in one experimental session lasting 1–2 h. Please note that the order of the experimental parts was important to firstly establish individual most ticklish body parts before testing for ticklishness suppression.

#### (i) Part I: tickling body parts

The purpose of this experiment was to characterize the tickle response pattern at different body parts and examine the resulting subjective experience of ticklishness. Tickling occurred at the head, neck, armpit, lateral trunk and plantar foot. The ticklee was instructed to position her/himself in the following way: both arms up (such that the tickler has access to armpit and trunk), sitting straight on a chair and having the right foot in a plantar-flexed/supine position (toes down) to enable tickling of the sole of the foot. Each body part was tickled five times at randomized sequence known to the tickler via a screen display outside the ticklee's field of vision. Intervals between tickling events varied across participants and were on average 9.54 s (s.e.m. = 1.433 s).

#### (ii) Supplementary part: reaction time task

The aim here was to obtain reaction times to non-ticklish tactile stimuli to be compared to ticklish vocal latencies observed in the first experiment following tickling at the most ticklish body part. Participants were tapped on their right shoulder, five times in total and instructed to respond to a tap with saying ‘Yes’ as quickly as possible once they felt the tap. Ticklers were asked to vary the time point of tapping randomly, at intervals of their choosing, and to keep the contact short and distinct.

#### (iii) Part II: self-tickle co-applied with allo-tickling

The objective of this experiment was to investigate the effect of self-tickling co-applied with allo-tickling. Participants' most ticklish body part from experimental part I was determined individually according to reported average rating and tickled exclusively in this part (an exception to this represents the foot as the further completion of the task was difficult to achieve; in these instances, the second most ticklish body part was used). Ticklees were asked to execute one of the following actions simultaneous to allo-tickling at said body part: self-tickling with the left hand with direct self-touch on the ipsilateral or contralateral side to the allo-tickling, or a tickle motion with the left hand in close proximity, yet without direct self-touch on the ipsilateral or contralateral side to allo-tickling. Further details on the experimental conditions are provided in the Results section. As control condition, participants were asked to perform no motion, akin to tickling in part I. This allowed us to establish a new baseline of ticklishness in order to account for potential fatigue when testing for suppression effects. The self-touch sequence was randomized, and each condition repeated five times. The ticklee was informed by the experimenter which action to perform ahead of each tickle event and commenced doing so a few seconds prior to allo-tickling. The tickler was instructed to vary the time point of tickling onset slightly each time to introduce a level of uncertainty for the ticklee when this would occur.

### (d) Video analysis

#### (i) Touch onset determination

Post-session video analysis was conducted in ELAN 5.4 (Max Planck Institute for Psycholinguistics, The Language Archive, Nijmegen, Netherlands) for determination of touch onset by visual inspection. Touch onset was defined as first point of external touch by the tickler.

#### (ii) Facial action coding units

Based on the characterization of observable muscle movements by Ekman & Friesen [24], it is suggested that facial action units (AUs) 06 (cheek raiser) and 12 (lip corner pull) constitute a Duchenne smile that is a facial expression signalling amusement or pleasure. Here, the analysis focused on these two units reported to have strong mean occurrences during ticklish laughter [23,24]. Tracing of facial AUs was achieved using OpenFace opensource software (https://github.com/TadasBaltrusaitis/OpenFace) based on convolutional experts constrained local model algorithm and dynamic modelling for facial landmark detection and AU classification [25–27].

### (e) Thoracic circumference

The analogue voltage signal of a respiratory belt transducer was digitized at 32 kHz using a DigitalLynx SX interface and Cheetah software. Traces were digitally filtered with a bandpass Butterworth filter with a filter range between 0.01 and 20 Hz. Traces were aligned in Matlab R2018b (The MathWorks, Inc., MA, USA) with the video data by sending 0.2 Hz TTL pulses simultaneously to both the DigitalLynx SX acquisition system and an LED pulse that was visible in the videos.

### (f) Audio analysis

The audio signal was filtered in the time-domain using the open-source software Praat 6.01.03 with a broadband FFT-based spectrogram (5 ms, Gaussian window, pre-emphasis +6 dB/octave). Praat was further used for the extraction of maximal pitch during voiced vocalizations using the autocorrelational method and a ceiling of search range of 50 000 Hz for both male and female participants.

### (g) Response latencies and amplitudes

For each tickle event, we defined the response latencies and amplitudes of audio, thoracic circumference, AU06 and AU12 traces with respect to tickle onset. Using the time stamps of touch onset, the traces were segmented into touch-triggered response intervals of 1 s prior to touch onset (baseline) and 1 s post touch onset (response window). Note that, given our paradigm, some trials did not elicit ticklishness responses. This was reflected in an absence of signal modulation after tickle onset. Please see the electronic supplementary material for details on the definition for single trial modulation.

For selected trials of AU06, AU12 and thoracic circumference, response latencies were determined using the Matlab function findchangepts to find the time point of most significant change in mean and slope of the signal during the response window. In order to detect the earliest change point, a maximum of two change points was computed, and the first one taken as response latency. For the audio signal, response latencies were computed manually for each selected trial (that is, trials with audible exclamation). The onset of a vocalization was determined by visual inspection of the spectrogram, and defined as the time point of a marked rise of energy from background noise.

The amplitude of each of the four signals was determined in selected trials. This was defined as the difference of the maximum value during the response window from the mean baseline level. For thoracic circumference, the absolute maximum was taken. Finally, for each trial, the maximal pitch during voiced vocalization at the first laughter interval from touch onset was determined. Given large between-subject variability in signal magnitudes, amplitudes were normalized for each participant individually.

### (h) Statistical analysis

Given the different research questions and variables of interest in both phases, we ran several mixed effects analyses. We used Matlab's fitlme function to fit six linear mixed effects models, using maximum-likelihood estimation. In all models, we employed a model comparison approach to select the optimal random effect structure [28]. A detailed description of the mixed effects models and the model comparison approach are presented in the electronic supplementary material.

## 3. Results

We conducted an experiment in two parts to (I) establish a timeline and quantify body part dependent ticklishness responses and subjective ticklishness scores, and (II) investigate self-touch suppression during co-applied self-and allo-tickling.

During part I, we tickled subjects at different body parts and measured a variety of response variables described below. Subjects were ticklish under our experimental conditions and emitted audible vocalizations in 70.5% (s.e.m. = 9.1%) of trials, when touched at ticklish body parts (neck, armpit, trunk and foot). By contrast, tickling at the crown of the head (a non-ticklish body part) evoked laughter in only 18.2% (s.e.m. = 8.6%) of trials. Almost all laughter in response to head tickling was observed in one extremely ticklish subject. Tickling of the head was excluded from further analyses unless otherwise stated. Individual subject vocalization probabilities are shown in electronic supplementary material, figure S1.

### (a) Multimodal time series of the ticklish response

**Figure 1.**
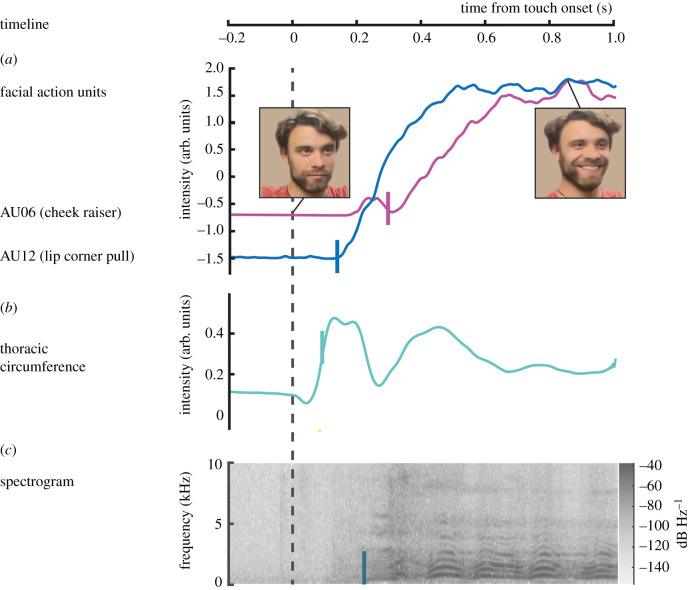
Multimodal time series of a tickle response. Time series of four physiological measures, shown exemplarily for one trial after tickling of the most ticklish body part (here, armpit) of participant 1. Data in all panels are shown relative to touch onset (dashed line). (*a*) Facial action unit AU12 (lip corner pull, blue trace) and AU06 (cheek raiser, purple trace). Video stills show the facial expression of the participant in that trial at the time of touch onset (left) and at the peak of the response at 0.86 s after touch onset (right). (*b*) Thoracic circumference. (*c*) Spectrogram of vocalization. Response latencies (solid vertical lines) were computed using the Matlab function findchangpts to find the most significant change points in mean and slope of individual traces within 1 s time-interval post touch onset.

To assess the physiological response to tickling, we measured changes in facial expression, thoracic circumference and vocalization as a function of time after tickle onset. Facial expression was quantified by facial action units AU06 (cheek raiser) and AU12 (lip corner pull), the occurrence of which in combination signals a joyous smile (figure 1*a*; [27]). Thoracic circumference measures expansion and contraction of the thorax and presumably reflects altered breathing in response to a tickle (figure 1*b*). Finally, vocalizations as visualized in the spectrogram (figure 1*c*), are defined as voiced and unvoiced audible exclamations (typically laughter).

**Figure 2.**
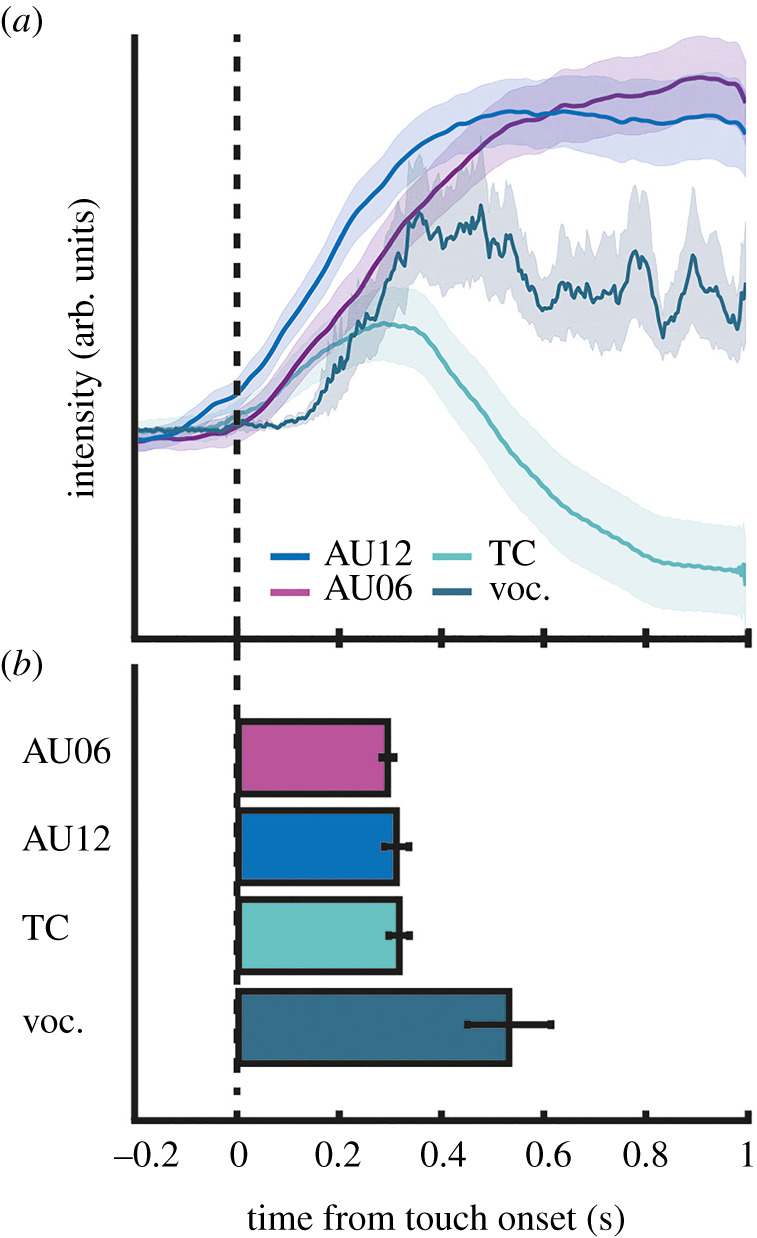
Average signals and response latencies of physiological measures. (*a*) Grand-average traces of the four signals shown in figure 1. Vocalization traces were computed via the Hilbert transform of the audio signal. For illustration purposes, single-trial traces were normalized by the subject-specific variance, and from each trial the average signal in the pre-touch period was subtracted. Mean signals are computed by pooling traces across participants, shaded areas show 95% confidence intervals. Only trials for which a response latency could be determined were included in the average. Please note that facial muscle activation (AU12) sometimes started prior to ticklish touch. Putatively, this is due to anticipatory effects of the tickle sensation. Although caution was taken to keep the exact time point of tickling unknown to the subjects, the general expectancy to be tickled may have sufficed on some occasions to elicit early responses. (*b*) Mean response latencies (and s.e.m.) of the four measures, shown in order of their mean.

We examined the temporal unfolding of these four parameters across all participants and all ticklish body areas (that is excluding the head) during a ticklish response. Mean traces of facial expression and thoracic circumference show a fast change after tickle onset, followed by delayed vocalizations (figure 2*a*). We determined for each measure the time point of most significant change of the signal after tickle onset, hereafter referred to as response latency (see methods for details). Mean response latencies of the four measures (figure 2*b*) show the following pattern: facial expression and changes in thoracic circumference co-occur at roughly 300 ms after tickle onset (AU06: *M* = 0.294 s, s.e.m. = 0.013; AU12: *M* = 0.316 s, s.e.m. = 0.025; thoracic circumference: *M* = 0.317 s, s.e.m. = 0.021), and vocalizations at approximately 500 ms after tickle onset (*M* = 0.534 s, s.e.m. = 0.083). Paired, two-sample *t*-tests showed that mean latencies did not differ between AU06 and AU12 (*t*_10_ = −0.69, *p* = 0.5), or between AU12 and expansion and contraction of the thorax (*t*_10_ = −0.171, *p* = 0.868), whereas vocalizations occurred significantly later than thoracic movement (*t*_9_ = −3.23, *p* < 0.05).

In a supplementary task, we compared vocal reaction times (saying ‘yes’) in response to a shoulder tap (a non-ticklish tactile stimulus) to ticklish vocalization latencies following tickling at most ticklish body areas as determined during part I. A paired *t*-test reveals that verbalization of ‘yes’ (*M* = 0.324 s, s.e.m. = 0.03) occurred significantly faster (*t*_44_ = −2.174, *p* < 0.05) than ticklish laughter (*M* = 0.456 s, s.e.m. = 0.067). We further note greater variability during laughter latencies than in the ‘yes’ condition suggestive of more similar general vocal response abilities among participants (results are shown in electronic supplementary material, figure S2).

### (b) The subjective experience of ticklishness

We assessed tickle intensity based on subjective ticklishness reports following tickling at all body areas (head, neck, armpit, lateral trunk and plantar foot). Participants gave self-rated assessments of a ticklish percept following each tickle event on a Likert-type scale from 0 (not ticklish) to 10 (highly ticklish). The subject-wise and population distribution of rating per body area is shown in electronic supplementary material, figure S3. A repeated measures ANOVA shows significant differences in rating per body area (*F*_4,10_ = 11.4, *p* = 0.001) with nonsignificant interaction of subjects and body part (*F*_40,10_ = 0.72, *p* = 0.778). Bonferroni-corrected *post hoc* analysis reveals that head tickling (*M* = 0.582, s.e.m. = 1.539) is rated less ticklish than armpit (*M* = 5.109, s.e.m. = 1.297), trunk (*M* = 5.473, s.e.m. = 0.884) and foot (*M* = 5.218, s.e.m. = 0.868) tickling. Neck tickling (*M* = 3.636, s.e.m. = 1.484) does not differ significantly from head tickling or other body areas.

Next, we examined the relationship between the subjective experience of ticklishness and the four physiological responses we measured, that is thoracic circumference, facial action unit AU06, facial action unit AU12 and vocalization. From each measure, we considered two parameters as potential explanatory variables of subjective experience: first, response latency (as described above), and second, amplitude, defined as the largest signal difference between post-touch and pre-touch baseline period. For vocalization amplitude, we differentiated between the absolute signal amplitude (i.e. the loudness of the response), and its frequency (i.e. its pitch). We fitted two linear mixed models, one with the response latency and one with response amplitude of the measures as explanatory variables.

**Figure 3.**
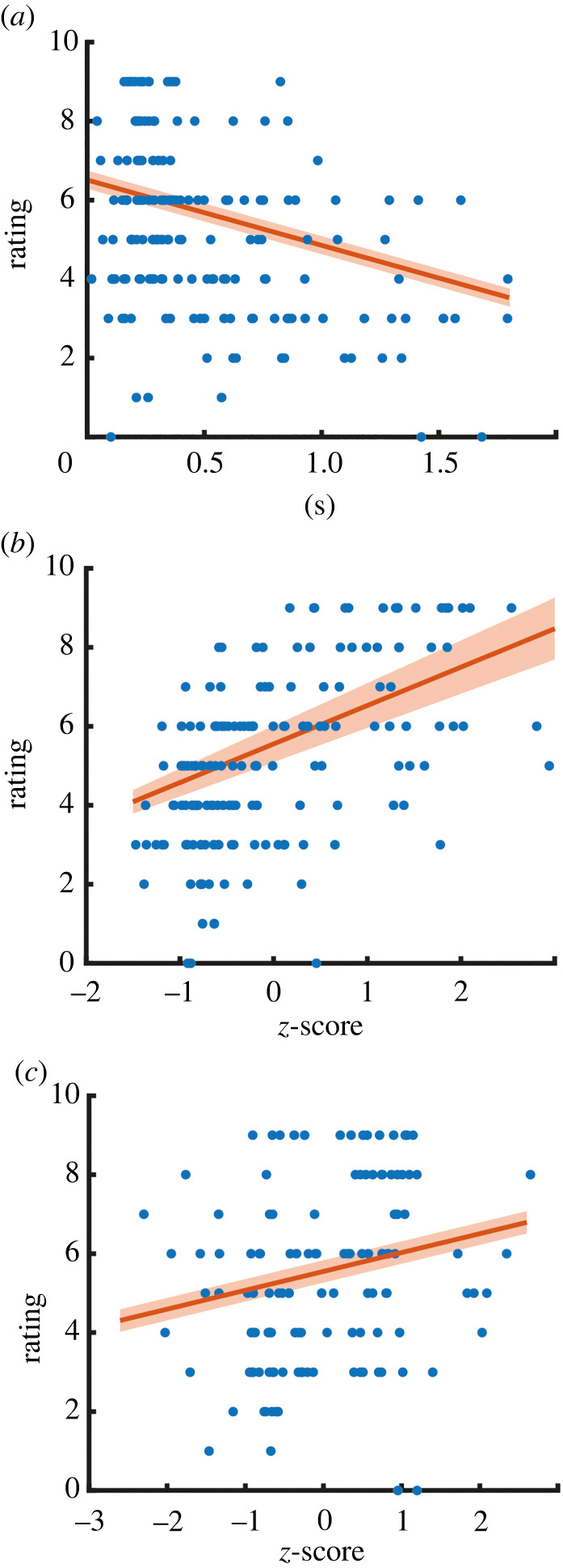
Properties of the vocal response predict ticklishness ratings. Mixed effects models assessing the effects of response latency and intensity on rating found (*a*) vocalization latency (*p* < 0.0001), (*b*) vocalization amplitude (*p* < 0.001) and (*c*) pitch (*p* < 0.001) to significantly predict subjective rating. Blue dots show single trial ratings, red lines show the average model prediction, and the shaded area shows the standard error.

As shown in figure 3*a–c*, the three parameters of vocalization response (latency, loudness and pitch) significantly correlated with subjective ticklishness scores. Specifically, an increase of 1 s in vocalization latency resulted in a decrease of rating by approximately 1.6 points (*β* = −1.61, *p* < 0.0001). Further, a 1 s.d. increase in vocalization amplitude resulted in an increase of rating by roughly 1 point (*β* = 0.97, *p* < 0.001). Finally, an increase of 1 s.d. of pitch resulted in about 0.5 points increase in subjective rating (*β* = 0.478, *p* < 0.001). Thus, with greater experienced ticklishness, laughter occurs faster, louder and more highly pitched. Thoracic circumference did not correlate with rating in either latency (*β* = −1.96, *p* = 0.14) or amplitude (*β* = −0.14, *p* = 0.35). The same holds true for AU06 (latency *β* = −1.62, *p* = 0.085; amplitude *β* = 0.06, *p* = 0.71) and AU12 (latency *β* = −0.076, *p* = 0.97; amplitude *β* = 0.11, *p* = 0.48).

### (c) Self-tickle induced suppression of the ticklish response

**Figure 4.**
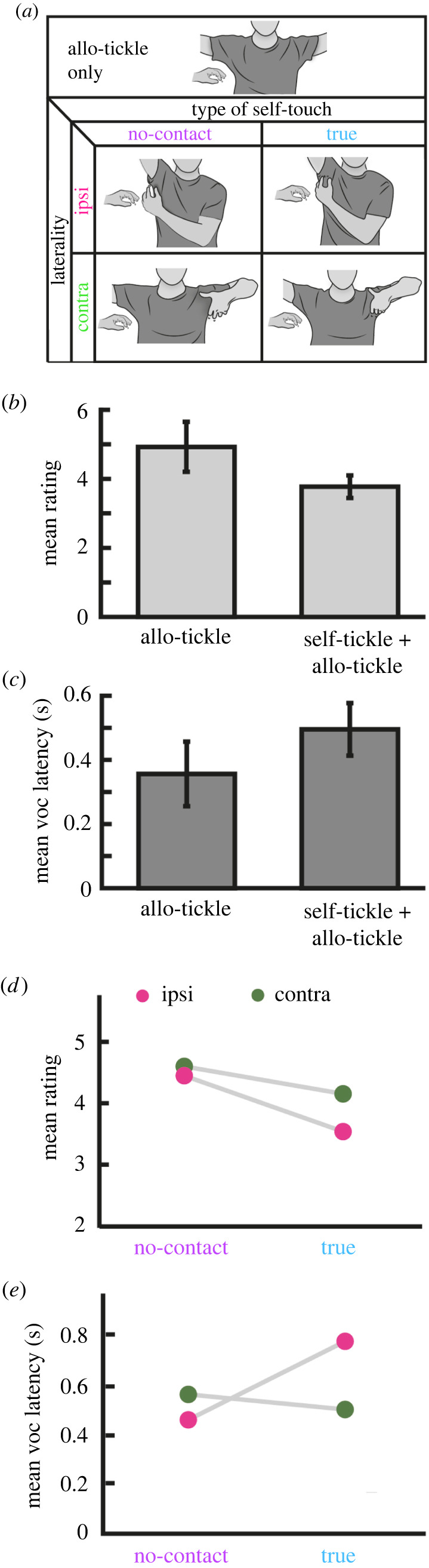
Effect of self-tickling on allo-tickle evoked ticklishness. Participants' most ticklish body part was identified from the average rating from part I and tickled exclusively for this part of the experiment (part II). (*a*) Ticklees always used their left hand to execute one of the following movements: no motion, akin to tickling in part I (allo-tickle only), self-touch in close proximity to the allo-tickle yet without making tactile contact (no-contact self-touch), or with tactile contact (true self-touch), each type of self-touch either on the ipsilateral or contralateral side to the allo-tickle (laterality). We show armpit-tickling exemplary. (*b*,*c*) Mean and 95% confidence intervals of subjective ticklishness ratings (*b*) and vocalization latencies (*c*), compared between allo-tickle only and co-applied self-tickle (all four conditions combined). Self-tickle + allo-tickle significantly reduced ticklish ratings (*p* < 0.0001) and delayed vocalization onset (*p* = 0.019) compared to sole allo-tickling. (*d*,*e*) Effect of type of self-touch and laterality on ticklishness rating (*d*) and vocalization latency (*e*). Rating was significantly reduced by true self-touch (*p* = 0.009), independent of laterality. Vocalization onset was delayed in the true self-tickle condition, only when the self-tickle occurred ipsilaterally (*p* = 0.003). Taken together, these results show that both subjective and physiological ticklishness responses are suppressed by self-tickling.

Findings in animals suggest diminished ticklish responses during co-occurring external tickling and self-touch [29]. Congruently, we predicted reduced ticklishness in humans during allo-tickling when coapplied with self-tickling in part II of the experiment. A graphical depiction of the paradigm is shown in figure 4*a*. We addressed the contributions of executing a tickle motion without direct touch (‘no-contact’ self-tickle) or with direct self-contact (‘true’ self-tickle), while alternating the targeted body side between the ipsilateral and contralateral side comparative to the side of allo-tickling for each self-touch type. Sole external tickling (allo-tickle) was repeated during this second part of the experiment.

In an initial step, we assessed how ticklishness response measures are affected by self-tickling *per se*, irrespective of body site or sensory consequence (that is, true and no-contact self-tickle on either side of the body combined) when compared with allo-tickling. We fitted two regression models to assess sole allo-tickle versus all other conditions with either rating or vocal latency as response variable. As shown in figure 4*b*, we find that participants gave significantly lower ticklishness scores (*β* = −0.6, *p* less than 0.0001) during self-tickling co-applied with allo-tickling (*M* = 3.769, s.e.m. = 0.611) compared to sole allo-tickling (*M* = 4.973, s.e.m. = 0.683). As shown in figure 4*c*, vocalization latencies increased significantly (*β* = 0.06, *p* = 0.019) during self-tickling (*M* = 0.473 s, s.e.m. = 0.129) when contrasted with allo-tickling (*M* = 0.37 s, s.e.m. = 0.105). Finally, participants were significantly less likely (*β* = −0.335, *p* < 0.05) to vocalize during self-tickle trials (*M* = 60%, s.e.m. = 10.4%) than during allo-tickling (*M* = 74.5%, s.e.m. = 11.5%). Individual subject data are shown in electronic supplementary material, figure S4.

Next, we addressed the specific contributions of tactile versus proprioceptive signalling (type of self-tickle), either within or across body hemispheres (laterality), and interactions thereof on ticklishness inhibition. As outlined above, we considered both vocal response latency and rating as response variables. We fitted two mixed effects models (one with vocal response latency and the other with ticklishness rating as response variables), each with type of self-tickle (true versus no-contact self-tickle on either side of the body), laterality (ipsilateral versus contralateral combining true and no-contact self-tickle events) as well as their interaction as main effects. Data from two participants were excluded from the vocal response latency model due to a lack of vocalization throughout this part of the experiment.

As shown in figure 4*d*, ratings were significantly reduced as a function of type of self-touch (*β* = −0.271, *p* = 0.008), with participants giving significantly lower ratings during true self-tickling (*M* = 3.5, s.e.m. = 0.676) compared to no-contact self-tickling (*M* = 4.039, s.e.m. = 0.561). Conversely, laterality of self-tickling was found not to be significant (*β* = −0.153, *p* = 0.137). That is, ratings were not affected by whether the self-tickle was carried out on the ipsilateral (*M* = 3.618, s.e.m. = 0.654) or contralateral (*M* = 3.92, s.e.m. 0.603) side. Finally, no significant interaction of type of self-tickle and laterality (*β* = −0.093, *p* = 0.367) was detected.

We found a significant interaction of type of self-tickle and laterality (*β* = 0.078, *p* = 0.003) on vocalization latency during self-tickling (figure 4*e*). That is, ipsilateral self-tickle resulted in the largest increase in vocalization latency, only if such action was carried out with true self-touch (*M* = 0.698 s, s.e.m. = 0.19). By contrast, contralateral self-tickle with true self-touch had no such effect (*M* = 0.468 s, s.e.m. = 0.139), neither did contralateral (*M* = 0.467 s, s.e.m. = 0.134) or ipsilateral self-tickle (*M* = 0.398 s, s.e.m. = 0.112) with no-contact self-touch. While the model reveals a significant main effect of type of self-touch on vocalization latency (β = 0.0526, *p* = 0.04) with later incurred vocalization during true self-tickling (*M* = 0.529 s, s.e.m. = 0.145) than no-contact self-tickling (*M* = 0.431 s, s.e.m. = 0.121), this effect should be treated with caution in light of the significant interaction of true ipsilateral self-tickle. No main effect of laterality was found (ipsilateral, *M* = 0.5 s, s.e.m. = 0.131; contralateral, *M* = 0.449 s, s.e.m. 0.13). Thus, vocalization latency is delayed during self-tickle action when it is carried out with true self-touch at the same side to allo-tickling. Taken together, the results of both models suggest that both subjective and physiological ticklishness responses are suppressed by self-tickling. Individual subject data is presented in electronic supplementary material, figure S5.

## 4. Discussion

Ticklishness in humans and its subjective and physiological features are not well understood. In particular, most research on ticklishness so far has disregarded the distinction between the two types of ticklish sensations, namely knismesis and gargalesis. Here, we performed a systematic assessment of the response pattern characterizing human gargalesis in a controlled environment. We find that (i) the first physiological responses to ticklish touch occur fast after approximately 200 ms, while the vocalization onset occurs substantially later, and that (ii) self-assessment of the intensity of a tickle correlates uniquely to acoustic properties of ticklish laughter. Furthermore, we investigated the effects of self-tickle on ticklishness and find that (iii) self-tickling when co-applied with allo-tickling reduces ticklishness ratings and delays vocalization onset.

### (a) The timing of the human tickle response

We show a temporal pattern of a ticklish response with notably faster response latencies than previously reported for tickle stimulation evoking knismesis [7]. Following ticklish touch onset, facial muscle movements and altered expansion and contraction of the thorax (putatively breathing) emerge in close temporal proximity within 300 ms and conclude in vocalizations (laughter) at around 500 ms. Interestingly, ticklish laughter occurs considerably later (approx. 170 ms) than ‘normal’ tactile processing reaction times or preparatory processes as part of a ticklish response. As elicitation of gargalesis requires vigorous finger movement, i.e. multiple rhythmical touches, laughter may need a longer time to be evoked than other behavioural responses. In addition, widespread cortical and subcortical processing during ticklish laughter may further contribute to delayed vocalizations. Greater involvement of the limbic pathway was identified during ticklish laughter compared to voluntary initiated laughter, and ticklish laughter suppression using functional imaging [30]. Observationally, we further note that ticklish vocal latencies were more variable than vocal latencies in the cued speech paradigm. While it cannot be excluded that differences in variability reflect differing states resulting from task demands, differences in variability may arise from differences in cortical or subcortical processing. Individual ticklish differences may be therefore more dependent on activation of the limbic system (e.g. high or low fight-or-flight network activation) that might in turn lead to high ticklishness in some and low ticklishness in others.

### (b) Physiological mechanisms

We find that greater subjective ticklishness (higher self-rating scores) co-occurs with heightened intensity, pitch and speed of laughter following ticklish touch. Assessments of physiological changes in thoracic circumference or socioemotional signalling expressed in joyous facial expressions do not show results to the same effect. Intriguingly, coupling of an intact auditory system and play behaviour in rats was also demonstrated [31]. Here, deaf animals showed significantly reduced pinning behaviour with unaltered dorsal contact indicative for lessened play behaviour with spared motivation for play. Deprivation of other sensory modalities such as sight or vibrissae removal did also reduce pinning, albeit to a non-significant degree. Further, particular 50 kHz calls are used to signal and promote play behaviour [32], and only about half of playful attacks are observed in pairs of devocalized rats compared to normal rats [33]. As our data indicate a striking correlation between auditory properties of ticklish laughter and subjective ticklishness ratings, we wonder if this relation is only correlational or if laughter also informs ourselves of how ticklish a tactile event was, i.e. if ‘hear’ rather than only ‘feel’ ticklishness.

### (c) Self-tickle suppression

Self-generated tickle motion when co-executed with allo-tickling induces ticklishness suppression with reduced self-rating and delayed vocalization onset when compared to allo-tickling. Such ticklishness suppression is greatest for (i) self-reported ticklishness when the self-produced movement results in tactile sensation, and for (ii) vocalization latency if the self-touch is carried out with true self-touch within the same body hemisphere as external stimulation. This is indicative for a more specific contribution of tactile sensory processing during self-touch suppression than previously reported [34,35].

Classical approaches evoking efferent motor command copies pertain that sensory attenuation for self-action requires no or minimal discrepancy between action and sensory feedback [5]. Under this model, externally elicited touch (i.e. a tickle) weakens accurate sensory prediction and thereby gives rise to a prediction error that induces ticklishness. By contrast, our data suggest a more general inhibitive process resulting in attenuation of temporally tuned external tickle stimulation. As such, our results tie in well with a growing body of evidence questioning the necessity of predictive precision in sensory attenuation [36–38]. In rats, convergent behavioural and electrophysiological data from our group indicate sensory modulation of self- and external touch based on balancing of inhibitory and excitatory processes during coinciding stimulation [29]. We propose that touch elicits afferent cortical excitation (independent of agency) and self-touch causes cortical inhibition (independent of afferent excitation). During co-applied self- and externally generated stimulation, somatosensation is determined by the weighting of excitatory/inhibitory signalling with self-touch evoked inhibition exceeding excitation. Thus, diminished tickle sensation arises from the sensory consequence of self-touch (tactile afferent processing) rather than movement *per se*. Such an explanation serves well to account for heightened ticklishness inhibition during direct tactile self-stimulation comparative to no-contact (proprioceptive) self-tickling. Further, stronger inhibition on the ipsilateral side as allo-tickling is predicted by this account, as we show for vocalization latency. However, coinciding detection as outlined here does not readily explain reduced somatosensation due to proprioceptive signalling that we observe when participants execute a tickle motion without direct contact. As suggested elsewhere [39], precise proprioceptive and somatosensory perception may preclude self-generated action, and temporary withdrawal of attention (precision) from sensory evidence is a prerequisite for movement to unfold. Reduced ticklishness may then be viewed as a necessity of movement execution (irrespective of tactile/no-contact self-tickle). In order to further elucidate the mechanisms of ticklishness attenuation, it would be of interest to investigate whether motionless tactile touch (i.e. resting one's hand at a body area that is simultaneously tickled by another person) results in a reduction in somatosensation. Imaging experiments on such behavioural paradigm as explored here (i.e. combined self- and allo-touch) might further elucidate processes associated with self-tickle suppression.

## 5. Conclusion

We conducted a systematic investigation of the human tickle response. Our data show that physiological responses to tickling occur within half a second after tickle onset, considerably faster than previously reported. Furthermore, loudness, pitch and latency of ticklish vocalizations are directly related to the subjective experience of ticklishness, suggesting that ticklishness may be a multisensory percept. Finally, we show that self-tickling co-applied with allo-tickling results in a suppression of both the physiological and subjective ticklishness response. We suggest that this effect can be understood as broad sensory attenuation of sensory inputs that temporally coincide, and highlight the potential role of tactile processing therein.

## Data Availability

The data and analysis scripts supporting this article have been uploaded as part of the supplementary material [40].
